# Cases Intended to Illustrate the Contagious Nature of Some Forms of Croup

**Published:** 1826-01

**Authors:** James Sym


ART. III.—*Cases intended to illustrate the Contagious Nature of some Forms of Croup.* By JAMES SYM, Esq. (Communicated by Dr. G. GREGORY.)

EARLY in the spring of 1824, ulcerated sore-throat, accompanied in some instances by scarlatina, became prevalent in this neighbourhood. In general it did not prove fatal; but a number of cases became complicated with croup, and of such cases I only met with one which terminated favourably. The disease seemed to be contagious among children; and I was even called to an old man, an Elder in the church, who died of a similar affection. He had been very obliging in visiting the sick, for religious purposes; and he had spent a considerable time in an apartment, in which an infant was dying of this disease. Two days after the death of the infant, he was seized with sore-throat; and when I saw him, on the third day of his illness and twenty-four hours before his death, his fauces were much ulcerated, and he laboured under the most torturing dyspnœa I have almost ever witnessed. He had dreadful approaches towards suffocation, which seemed to be kept off for a season by the expectoration of large quantities of mucus during his struggles.

This was the only instance of an adult being affected with the disease that fell under my observation; and it seemed to differ from the affection in children in this respect only, that the secretion from the tracheal membrane consisted of viscid mucus, instead of coagulating lymph.

In the infants, the symptoms of croup were most decided; the affection of the fauces being in many cases so trifling, that it had not been attended to by their mothers, until I detected the ulcers, upon examination, after the accession of croup had commenced. In one family, consisting of three children, who were successively attacked by the disease, two died and one recovered; the only cure of the complicated affection that occurred in my practice. Having examined the windpipe of one of these children after death, I found it filled with a thick, firm, lymphatic tube, which extended into the bronchial ramifications. In the case which terminated favourably, the ulcers were getting better when the croup commenced; and to this circumstance I attribute the efficacy of the remedies in operating a cure.

From what I observed, I concluded that the ulcerated sore-throat, which appeared to be closely allied to scarlatina, constituted the contagious part of the disease; and that the croup was occasioned by an extension of the inflammation at the margin of the ulcers into the rima glottidis, and from thence along the mucous membrane of the trachea. This idea seems to derive confirmation from your cases; and, if further supported by the observation of other practitioners, it may account for the contagious character of a certain species of croup.

*Kilmarnock, Ayrshire; 14th Oct.* 1825.

